# Age-Based Variations in the Gut Microbiome of the Shennongjia (Hubei) Golden Snub-Nosed Monkey (*Rhinopithecus roxellana hubeiensis*)

**DOI:** 10.1155/2021/6667715

**Published:** 2021-03-12

**Authors:** Lijuan Yao, Xiang Li, Zutao Zhou, Deshi Shi, Zili Li, Shangfei Li, Hui Yao, Jingyuan Yang, Huiliang Yu, Yuncai Xiao

**Affiliations:** ^1^State Key Laboratory of Agricultural Microbiology, College of Veterinary Medicine, Huazhong Agricultural University, Wuhan, 430070 Hubei, China; ^2^National Center for International Research on Animal Genetics, Breeding and Reproduction, Huazhong Agricultural University, Wuhan, 430070, China; ^3^Key Laboratory of Agricultural Animal Genetics, Breeding and Reproduction of Ministry of Education, College of Animal Science and Technology, Huazhong Agricultural University, Wuhan, 430070, China; ^4^Hubei Province Key Laboratory of Conservation Biology of Shennongjia Golden Monkey, Hubei Conservation and Research Center for the Golden Monkey, Muyu, Shennongjia, 442411 Hubei, China

## Abstract

The gut microbiota represents a source of genetic and metabolic diversity of a complex polymicrobial ecosystem within its host. To investigate age-based variations of the gut microbiota among Shennongjia golden snub-nosed monkeys (*Rhinopithecus roxellana hubeiensis*), we characterized the microbial species in fecal samples from 18 Shennongjia golden snub-nosed monkeys evenly pooled into 3 aged groups (Group 1, 1-3 years; Group 2, 5-8 years; Group 3, above 12 years) in Shennongjia, Hubei Province, China. Genomic DNA was extracted from fecal samples, and the 16S rRNA gene V4 region was sequenced using the Illumina high-throughput MiSeq platform PE250. A total of 28 microbial phyla were identified in the gut microbiome of these monkeys with the ten most abundant phyla (i.e., Firmicutes, Bacteroidetes, Verrucomicrobia, Spirochaetes, Tenericutes, Proteobacteria, Planctomycetes, Fibrobacteres, Cyanobacteria, and Euryarchaeota). A total of 1,469 (of 16 phyla and 166 genera), 1,381 (of 16 phyla and 157 genera), and 1,931 (of 19 phyla and 190 genera) operational taxonomic units (OTUs) were revealed in Groups 1, 2, and 3, respectively, with Group 3 containing the most diverse groups of OTUs as revealed by the species relative abundance clustering analysis. These results suggest that the gut microbiota in these monkeys maintain a dynamic status, starting from the early developmental stages of life with the species relative abundance increasing with age. This is the first study to comprehensively characterize the gut microbiota and provide valuable information for monitoring the health and nutritional needs of this endangered primate at different ages.

## 1. Introduction

The snub-nosed monkeys comprise the genus *Rhinopithecus*, representing a radiation of Asian colobines including *R. roxellana*, *R. bieti*, *R. brelichi*, *R. avunculus*, and *R. strykeri* [1, 2]. Colobine monkeys are generally characterized by a specialized set of digestive adaptations enabling them to consume difficult-to-digest foods. Their enlarged and multichambered stomachs with undeveloped cecum and colon contain a poor capability in grinding foods mechanically, while the forestomachs in golden snub-nosed monkeys are harboring microorganisms to help their hosts ferment foods with large fiber content. The ability to ferment generally indigestible foods makes the golden snub-nosed monkeys more folivorous, often showing gastrointestinal problems in captivity [3].

The population of golden snub-nosed monkeys in the Shennongjia National Nature Reserve, Hubei, China, is recognized as *R. roxellana hubeiensis* (commonly known as Shennongjia or Hubei golden snub-nosed monkeys) and is one of the endangered colobine species listed in the Appendix I of the Convention on International Trade in Endangered Species [4]. Its conservation and scientific significance has been emphasized recently based on its restricted distribution, high genetic divergence, and similar evolutionary status as its sister subspecies *R. roxellana roxellana* [5], which is commonly known as Sichuan golden snub-nosed monkeys. The Shennongjia golden snub-nosed monkeys generally live in a group composed of several small family (reproductive) units responsible for producing infants with one male individual responsible for the safety of the entire group [6]. They normally mate from August to October and give birth between March and June each year [7]. They are only found within the Shennongjia National Nature Reserve, Hubei Province, China, in the temperate deciduous and coniferous forests at the elevation of ~2200 m [7–10]. Their main foods contain both the self-feeding seasonal materials (e.g., leaves, flowers, buds, seeds, lichens, and fruits) and provisioned seasonal fruits (e.g., apples and peaches), sweet potatoes, and peanuts. At the Shennongjia National Nature Reserve, the seasonal fruits and peanuts are provided to the monkeys by breeders 3 times a day in spring, summer, and autumn, while sweet potatoes and peanuts are provided in winter only. Field studies from several sites across China have documented that the self-feeding foods account for ~74%, 68%, ~81%, and~53% of their total foods in spring, summer, autumn, and winter, respectively [7, 9].

The intestinal tract is colonized by a large number of active cells and a complex gut microbiota. For example, the human intestinal tract is generally colonized by up to 100 trillion microorganisms representing ~300-1000 species [11]. The gut microbiota is predominantly composed of bacteria forming an important part of the body's normal ecosystem [12]. The gut microbiota actively participates in host's nutrition metabolism and immune regulation and plays a key role in immunological homeostasis, food digestion, protection against pathogenic bacteria colonization, fat accumulation, reproduction, and brain behavior and development [13]. Under normal circumstances, the gut microbiota exists in a dynamic balance and serves to activate host immune system as well as regulate host metabolism. However, any perturbations in gut microbiota can result in intestinal disorders [14]. The effects of treatment to alter the gut microbiota of patients with metabolic and digestive diseases have been investigated extensively in recent years [12, 15, 16].

Studies have shown that the gut microbiota begins to establish prior to birth and develops rapidly with age [17]. Furthermore, the development of the gut microbiota is closely related to the host health and certain health problems, such as irritable bowel, gastroenteritis, and overweight status [18]. The gut microbiota of golden snub-nosed monkeys is likely to play an essential role in nutrition, development, metabolism, pathogen resistance, and regulation of host immune responses. Despite the importance of the composition and development of gut microbiota, studies to characterize gut microbiota in Shennongjia golden snub-nosed monkeys are sparse [3, 19–21]. Therefore, the comprehensive characterization of gut microbiota in Shennongjia golden snub-nosed monkeys of different ages will be essential for monitoring related intestinal diseases in these monkeys.

Human gut microbial development from infancy through youth has been thoroughly described [22]. The association between age and both structural and functional changes in gut microbiota has been well established based on multiple lines of genomic and molecular evidence [23]. For example, aging is accompanied by major alteration in the gut microbial composition, causing various disorders in the gastrointestinal tract [24, 25], as demonstrated by the loss of microbial stability in older adults and subsequently the reduced appetite, digestion, absorption, and ultimately malnutrition [26–28]. However, little is known about the differences between the gut microbiota among different primate species. In general, sampling a broad array of fecal samples of healthy humans representing different ages provides a great opportunity to discover the evolution of human gut microbiomes with the developmental stages in life. For example, studies have shown that the development of gut microbiota is related to age in some species with the change of relative abundances but not the types of the major bacterial species, while the gut microbiota changes considerably between 6 and 12 months of life and then develops to a profile characteristic of the adult microbiota in a state of dynamic equilibrium [22, 29].

The high-throughput sequencing technology has been applied to characterize gut microbiota in a few species of golden snub-nosed monkeys [3, 19–21]. With the availability of these well-established methodologies and well-documented characterization of microbial community compositions in the gut microbiome of these closely related golden snub-nosed monkeys, the objectives of our study were to characterize the bacterial colonization and composition of the intestinal tract in healthy Shennongjia golden snub-nosed monkeys with ages of 1 to 12 years and the development of their gut microbiota using high-throughput sequencing of the 16S rRNA gene of the gut microbiome. Previous studies showed that the microbial diversity in the gut microbiota of humans increases as they age [30]; therefore, we predicted that the microbial diversity in the gut microbiota of Shennongjia golden snub-nosed monkeys would increase as their developmental ages. Our results provide valuable information for monitoring the health and nutritional needs of this endangered primate.

## 2. Materials and Methods

### 2.1. Ethics Statement

This study was performed in accordance with the Guide for the Care of Animals of the Ministry of Health, China. The protocol was approved by the Hubei Conservation and Research Center for the Golden Monkey (Organization Code Certificate: 42218045-1). The design and analysis of this study were performed in accordance with the Animal Research: Reporting In Vivo Experiments (ARRIVE) guidelines and the American Society of Primatologists Principles for the Ethical Treatment of Non-Human Primates. All fecal samples were collected from the Shennongjia golden snub-nosed monkeys with permission approved from the Hubei Conservation and Research Center for the Golden Monkey including the number of people and the time allowed to collect samples.

### 2.2. Fecal Sample Collection of Shennongjia Golden Snub-Nosed Monkeys

A group of ~69 Shennongjia golden snub-nosed monkeys observed in this study were located in a wildlife reserve of the Hubei Conservation and Research Center for the Golden Monkey in the Shennongjia National Nature Reserve (31°21′–31°37′N and 110°03′–110°34′E), Hubei Province, China. The group included ~23 (1-3 years), 20 (5-8 years), and 26 (above 12 years) golden snub-nosed monkeys, all staying under natural living conditions with their feeding primarily provided by the breeders at the Hubei Conservation and Research Center for the Golden Monkey in the Shennongjia National Nature Reserve.

During this study, a total of 18 fecal samples from 18 (out of 69) Shennongjia golden snub-nosed monkeys were collected based on the availability for high-throughput sequencing of the V4 regions of 16S rRNA gene. The 18 monkeys were equally divided into three groups, including Group 1 containing monkeys of 1-3 years old, Group 2 with monkeys of 5-8 years old, and Group 3 with monkeys above 12 years. This grouping strategy was based on the knowledge that Shennongjia golden snub-nosed monkeys reach sexual maturity at ~5-7 years with an average life span of ~12 years [8]. All of the 18 Shennongjia golden snub-nosed monkeys studied in this study were healthy when the fecal samples were collected. The fecal samples were collected immediately after the excretion from monkeys by wearing sterile disposable gloves in order to avoid cross contamination. Sterility was maintained during the collection of fecal samples which were handled gently to reduce damage to the surface of the samples. The fecal samples were immediately placed in a 15 mL centrifuge tube and stored at −80°C for future experiments. No monkeys were sacrificed during the experiment.

### 2.3. DNA Extraction and High-Throughput Sequencing of 16S rRNA Gene

Genomic DNA was extracted from fecal samples using a stool DNA kit (Omega, BioTek Inc., USA) and stored at −20°C for further experiments. The V4 region of 16S rRNA gene was amplified using primers 515F (forward: GTGCCAGCMGCCGCGGTAA) and 806R (reverse: GGACTACHVGGGTWTCTAAT). The reverse primer 806R contained a 6 bp error-correcting barcode unique to each of the 18 fecal samples. The mixture of the PCR contained 10 *μ*L of DNA template, 3 *μ*L of each primer, 15 *μ*L of Phusion Master Mix (20×), and ddH_2_O added to make a final volume of 30 *μ*L. PCR procedures were carried out as follows: 1 min at 98°C for denaturation, followed by 30 cycles of 10 s at 98°C for denaturation, 30 s at 50°C for annealing, and 30 s at 72°C for elongation, a final extension of 5 min at 72°C, and hold at 4°C. PCR products were purified with TruSeq PCR-Free DNA Sample Preparation Kit to make the high-throughput libraries and quantified by spectrophotometry using the NanoDrop (Thermo Fisher Scientific, Inc.) for normalization to a final concentration of 2 nmol/L. The library pool was paired-end sequenced with an Illumina MiSeq platform PE250 (Novogene Biotech Co., Ltd., Beijing, China).

### 2.4. Sequence and Data Analyses

Mothur (version 1.31.2) was used to process the 16S rRNA gene sequences following the MiSeq standard operating procedure (https://mothur.org/wiki/miseq_sop/) [31]. Specifically, Mothur was applied to trim, screen, and assign sequences to operational taxonomic units (OTUs), and further describe both the alpha and beta diversities of samples characterized by 16S rRNA gene sequences. Alternative software packages were applied to conduct relevant analyses. The effective sequences were assigned into OTUs based on the SSU rRNA database in SILVA 138 (http://www.arb-silva.de/) using a similarity cutoff of 0.03 (i.e., similarity of 97%) for the distance matrix and species classification generated based on DOTUR in Mothur [32]. The sequences were clustered using unweighted pair group method with arithmetic means (UPGMA) algorithm. To get the species information and its relative abundance distribution, both Ribosomal Database Project (RDP) classifier [33] and GreenGene database [34] were used to annotate and analyze the representative sequences in the corresponding OTUs. The samples were rarefied (i.e., normalization) to equal sequencing depths prior to further diversity analyses. The species richness and evenness in each sample and group were obtained by calculating the relative OTU abundance and the alpha diversity (i.e., observed species index, Simpson's diversity index, chao1 diversity index, ACE diversity index, and Shannon's diversity index). Species richness was presented using the Venn maps. For the beta diversity analysis, the principal coordinate analysis (PCoA) and the unweighted pair group method with arithmetic means (UPGMA) grouping based on weighted UniFrac distances were performed to compare the samples and identify the differences between the aged groups of monkeys. Results of PCoA were presented with 3 dimensions using JMP software (http://www.jmp.com). Based on UniFrac distance, significant difference among the three groups of samples was evaluated using AMOVA in the Mothur package (http://www.mothur.org/wiki/Amova). For both alpha and beta diversity analyses, the significant differences among samples and groups were evaluated using the Wilcoxon rank sum test in the Agricolae package. The Benjamini and Hochberg false discovery rate (FDR) analysis with corrected *P* values was used to evaluate the differentially abundant taxa [35]. The indicator analysis was performed to identify specific OTUs associated with each age group. Both *t*-tests in R package and Metastats (i.e., FDR corrected *P* values among groups based on the permutation test without assuming the normal distribution of underlying data, commonly known as the nonparametric *t*-test) [35] were performed to identify the microbial organisms represented differently among different samples and groups at both the phylum and genus levels.

## 3. Results

### 3.1. Sequence and Data Analyses

A total of 1,006,343 reads (each of 253 bp in size) from 18 samples (with an average of 55,908 reads ranging from 32,415 to 76,639 reads) were filtered by quality control tests. The sequence statistics of the 18 samples and three groups of monkeys were provided in Table S1. The sequence data is publicly available in the Sequence Read Archive database at the National Center for Biotechnology Information (https://www.ncbi.nlm.nih.gov/) with the accession numbers of SRR1585338, SRR1585339, and SRR 1585340 under the BioProject accession PRJNA261867.

The rarefaction curves obtained by plotting the number of operational taxon units (OTUs) with the number of reads for all fecal samples were used to evaluate the efficacy of sampling efforts (Figure S1). These rarefaction curves showed that the number of reads were sufficient to cover the majority of the 16S rRNA sequences in each sample, demonstrating the high complexity of the intestinal microbiota in Shennongjia golden snub-nosed monkeys as represented by the sloping of many curves at the mark of 30,000 reads and the high coverage obtained for each sample, while the cures of the relative abundance reflected the high degree of uniformity and richness in the 18 samples.

**Figure 1:**
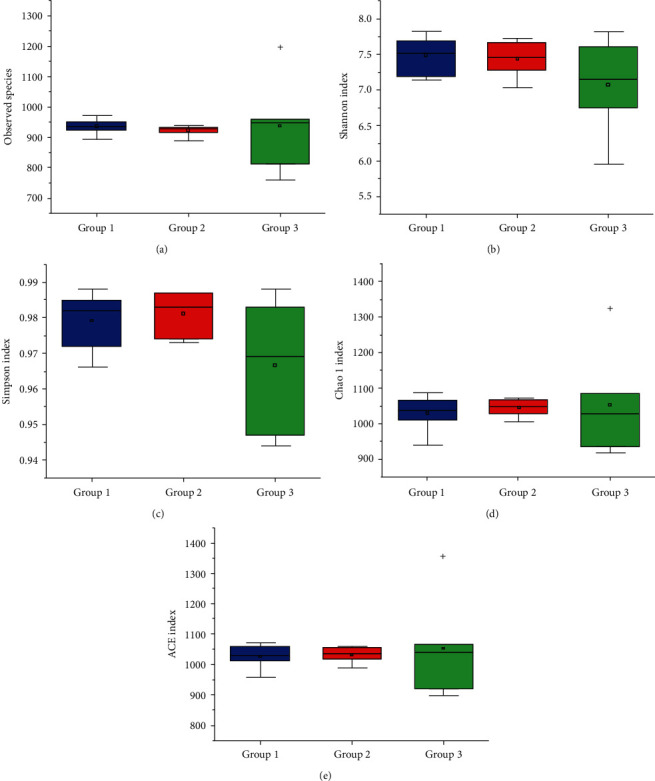
The alpha diversity indices including observed species index (a), Shannon's diversity index (b), Simpson's diversity index (c), chao1 diversity index (d), and ACE diversity index (e), presented in box plots of 18 fecal samples in three groups of Shennongjia golden snub-nosed monkeys.

A total of five alpha diversity indices (i.e., observed species, Simpson's diversity index, chao1 diversity index, ACE diversity index, and Shannon's diversity index) were used to assess the species richness and variation within each age group and individual sample (Figure 1). No significant difference was revealed for these diversity indices (*p* > 0.05). Similar to the rarefaction curves, the high Good's coverage indices ranging from 0.993 to 0.996 in all samples and three groups showed that the number of reads were sufficient to cover the majority of the 16S rRNA sequences in each sample.

### 3.2. Variations of the Microbial Community Compositions in Gut Microbiome of Three Groups of Shennongjia Golden Snub-Nosed Monkeys with Different Ages

**Figure 2:**
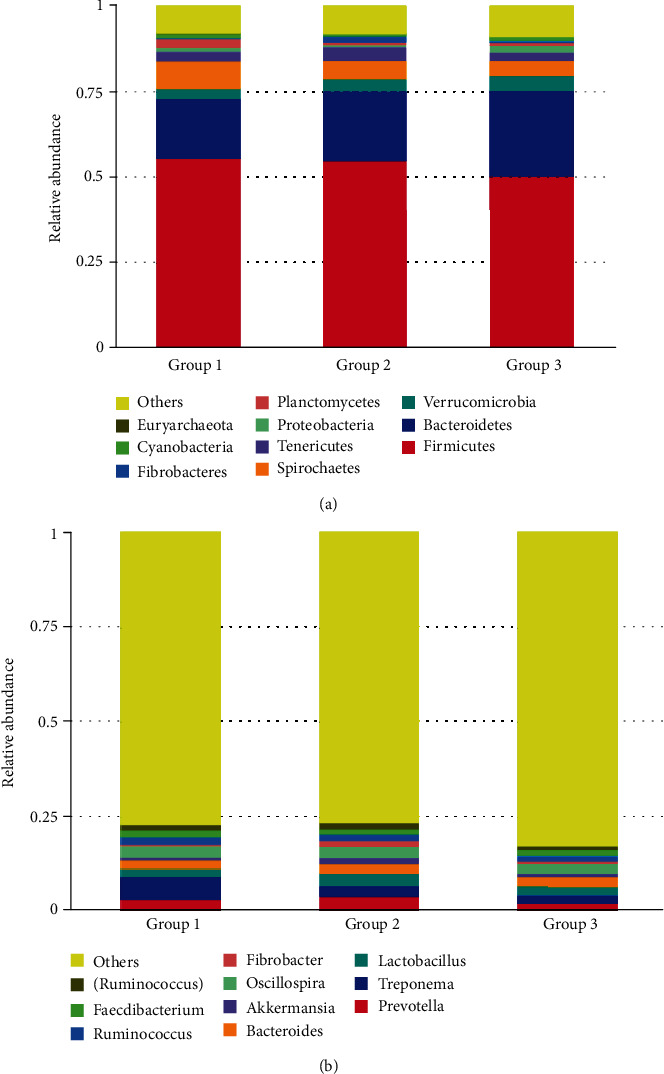
Relative abundance of the top 10 most abundant microbial phyla (a) and genera (b) identified in the fecal samples of three different aged groups of Shennongjia golden snub-nosed monkeys.

**Figure 3:**
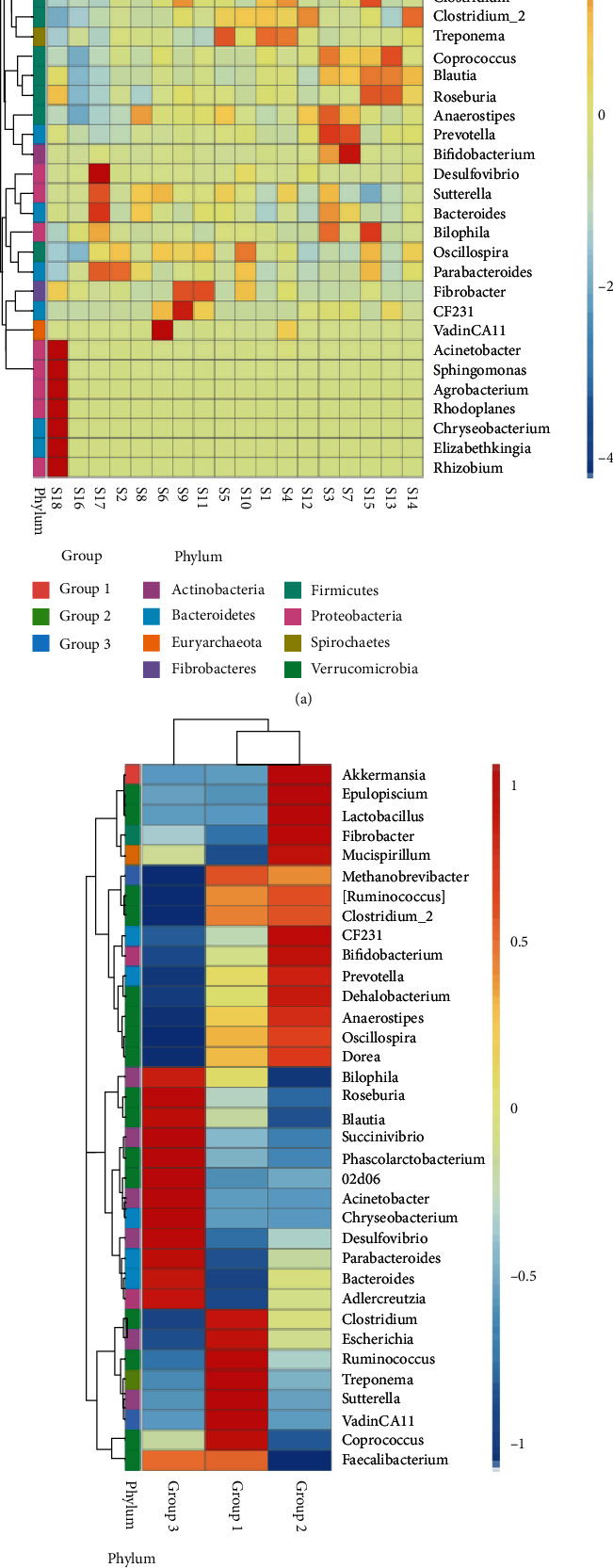
The relative abundance of top 35 most abundant microbial genera in the 18 fecal samples (a) of three groups (b) of Shennongjia golden snub-nosed monkeys based on the species relative abundance clustering and presented in heatmaps. Scaled bar represents log-transformed relative abundance.

A total of 1,469, 1,381, and 1,931 OTUs were assigned in 16, 16, and 19 phyla, and 166, 157, and 190 genera in Groups 1, 2, and 3 of fecal samples of Shennongjia golden snub-nosed monkeys, respectively. The sequences were classified into a total of 27 phyla of Bacteria and one phylum of Archaea. The top 10 most abundant phyla (9 of Bacteria and 1 of Archaea) and their proportions in each of the three groups are presented in Table 1. The relative abundances of these 10 phyla and the top 10 most abundant genera within each group and sample are presented in Figures 2 and 3, respectively. At the phylum level, Firmicutes and Bacteroidetes were the top two phyla and Euryarchaeota being the least abundant revealed in all three aged groups, while the proportions of other phyla largely varied, either increasing (e.g., Bacteroidetes, Verrucomicrobia, and Spirochaetes) or decreasing (Firmicutes, Planctomycetes, and Euryarchaeota), as the developmental stages (Table 1; Figure 2(a)). At the genus level, the most abundant genera in the three aged groups were *Treponema*, *Prevotell*, and *Oscillospira*, respectively (Figure 2(b)). Both Firmicutes and Bacteroidetes maintained as the top two most abundant phyla in each of the 18 samples, while five and two out of the top 10 most abundant genera belonged to phyla Firmicutes and Bacteroidetes, respectively, in the 18 individual samples (Figure S2).

The species relative abundance clustering method was used to analyze the variations in species of the top 35 most abundant microbial genera in each sample among three groups of monkeys (Figure 3). The sample #18 was notably revealed to show significantly more abundant in seven genera (*Acinetobacter*, *Sphingomonas*, *Agrobacterium*, *Rhodoplanes*, *Chryseobacterium*, *Elizabethkingia*, and *Rhizobium*) than other 17 samples (Figure 3(a)). A total of 8 genera (i.e., *Clostridium*, *Escherichia*, *Ruminococcus*, *Treponema*, *Sutterella*, *VadinCA11*, *Coprococcus*, and *Methanobrevibacter*) were significantly more abundant in Group 1 than those in Groups 2 and 3. A total of 14 genera (*Akkermansia*, *Epulopiscium*, *Lactobacillus*, *Fibrobacter*, *Mucispirillum*, *CF231*, *Bifidobacteria*, *Prevotella*, *Dehalobacterium*, *Anaerostipes*, *Oscillospira*, *Dorea*, *Ruminococcus*, and *Clostridium*) were significantly more abundant in Group 2 compared to Groups 1 and 3, while another 12 genera (*Bilophila*, *Roseburia*, *Blautia*, *Succinivibrio*, *Phascolarctobacterium*, *02d06*, *Acinetobacter*, *Chryseobacterium*, *Desulfovibrio*, *Parabacteroides*, *Bacteroides*, and *Adlercreutzia*) were significantly more abundant in Group 3 than those in Groups 1 and 2 (Figure 3(b)).

**Figure 4:**
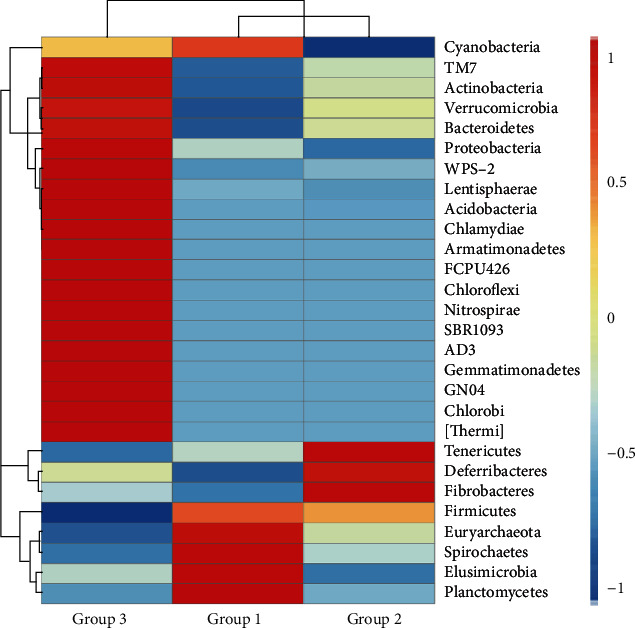
The relative abundance of a total of 28 microbial phyla identified in three aged groups of Shennongjia golden snub-nosed monkeys presented in a heatmap. Scaled bar represents log-transformed relative abundance.

At the phylum level (Figure 4), a total of 6 phyla (Planctomycetes, Elusimicrobia, Spirochaetes, Euryarchaeota, Firmicutes, and Cyanobacteria) were significantly more abundant in Group 1 than those in Groups 2 and 3, while phyla Tenericutes, Deferribacteres, and Fibrobacteres were significantly more abundant in Group 2 than those in Groups 1 and 3. A total of 19 phyla (TM7, Actinobacteria, Verrucomicrobia, Bacteroidetes, Proteobacteria, WPS-2, Lentisphaerae, Acidobacteria, Chlamydiae, Armatimonadetes, FCPU426, Chloroflexi, Nitrospirae, SBR1093, AD3, Gemmatimonadetes, GNO4, Chlorobi, and Thermi) showed significantly more abundant in Group 3 in comparison to Groups 1 and 2.

The microbial genera and phyla showing significant differences among the three aged groups of Shennongjia golden snub-nosed monkeys are presented in Tables 2 and 3, respectively. At the phylum level, the phylum Lentisphaerae was significantly more abundant in Group 3 than that in both Groups 1 and 2, while the phylum Planctomycetes was less abundant in comparison to Group 1. In general, the relative abundance of both phyla Lentisphaerae and Deferribacteres increased with age. At the genus level, three genera (*Mucispirillum*, *Treponema*, and *Succinivibrio*) were revealed to be significantly different between Groups 1 and 2 with *Mucispirillum*, while a total of 4 out of 8 genera (*Escherichia*, *Anaeroplasma*, *Clostridium*, and *Flexispira*) and 2 out of 7 genera (*Ruminococcus* and *Anaerostipes*) were significantly different between Groups 1 and 3, and Groups 2 and 3, respectively.

**Figure 5:**
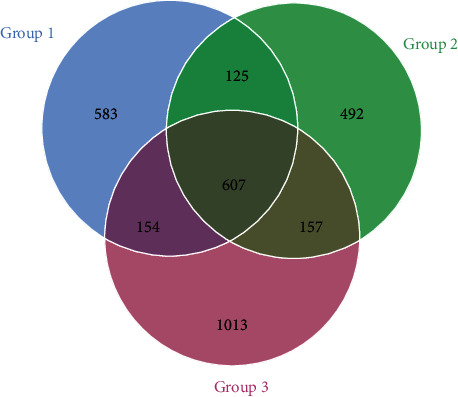
Venn map showing the distributions of common and unique operational taxonomic units (OTUs) among three aged groups of Shennongjia golden snub-nosed monkeys.

The numbers of the common and unique OTUs present in each of three aged groups were shown in the Venn map (Figure 5). A total of 607 OTUs were commonly presented in all three aged groups with Group 3 containing the most unique OTUs of 1,013, while Groups 1 and 2 contained a total of 583 and 492 unique OTUs, respectively. The individual sample #18 contained the largest number of unique OTUs of 439, while all other 17 individuals contained unique OTUs ranging from 7 (individual sample #16 in Group 1) to 37 (individual sample #6 in Group 1) (data not shown).

**Figure 6:**
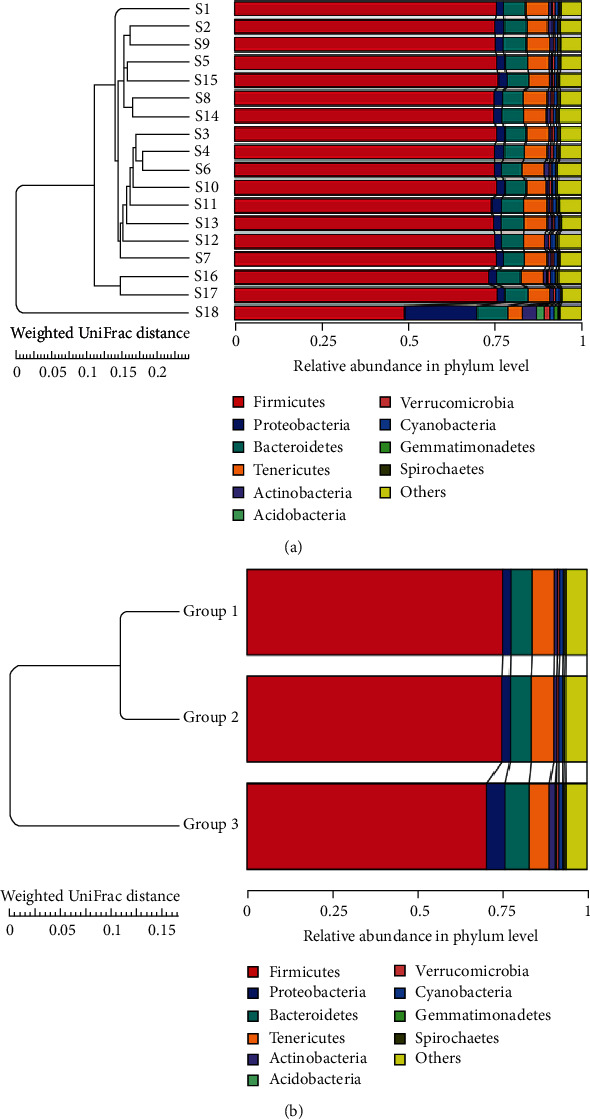
The UPGMA clustering of the microbial phyla derived from 18 individual samples (a) and three aged groups (b) of Shennongjia golden snub-nosed monkeys at phylum level based on weighted UniFrac distances.

The beta diversity analyses were performed to further characterize the variations of the microbial community compositions in the gut microbiome of three groups of Shennongjia golden snub-nosed monkeys. The UPGMA UniFrac clustering was used to compare the similarities among three aged groups and 18 individuals of Shennongjia golden snub-nosed monkeys at phylum level (Figure 6). The results showed that the six individual samples in each one of the three aged groups did not cluster together, indicating the difference between individuals at the same or similar developmental stages (Figure 6(a)). The individual sample #18 was not clustered with any other individual samples due to its high proportion of phylum Proteobacteria. The results also showed that the microbial phyla from Groups 1 and 2 clustered together containing the similar microbial compositions (Figure 6(b)).

**Figure 7:**
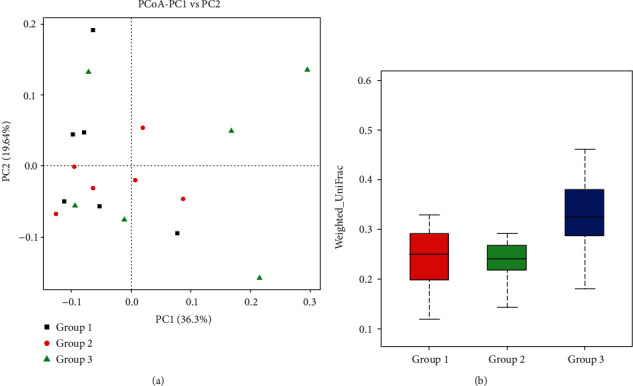
The principal coordinate analysis (PCoA) showing the PCoA plot (a) and the box plot (b) of three aged groups of Shennongjia golden snub-nosed monkeys based on weighted UniFrac distances.

The similarities among three aged groups of Shennongjia golden snub-nosed monkeys were further examined by PCoA based on the weighted variants of UniFrac (Figure 7). The results showed that Groups 1 and 2 shared a high similarity in the composition of the intestinal microbiota and a significant difference in comparison to Group 3.

## 4. Discussion

### 4.1. Biological Significance of Gut Microbiota in Shennongjia Golden Snub-Nosed Monkey

The gut microbiota has been widely studied in domestic animals, livestock, and several species of primates, including human, to help evaluate health conditions of its hosts [26, 36]. For example, the gut microbiota of captive common marmoset (*Callithrix jacchus*) and Chinese-origin rhesus macaques (*Macaca mulatta*) have been reported [37, 38]. Furthermore, the gut microbiota has been investigated in a few species of golden snub-nosed monkeys, including the Sichuan golden snub-nosed monkeys with diarrhea in comparison to the healthy monkeys [3, 19–21]. However, little is known about the gut microbiota of Shennongjia golden snub-nosed monkeys, particularly the dynamic status of the gut microbiome of age-related individuals of these monkeys. This information is extremely important for protecting and managing these endangered monkeys in both the wild and the captivity. With the recent availability of the whole-genome sequencing of Sichuan golden snub-nosed monkey, it is expected that the studies of this endangered species from many perspectives, e.g., the influence of genetics, diet, and environment on the biological functions of the gut microbial community, would be greatly facilitated [39].

In our study, the 16S rRNA sequences were analyzed based on 18 samples of three integrated age-based groups each containing six monkeys of 1-3, 5-8, and above 12 years, respectively. This grouping strategy allowed the appropriate assessment of microbial composition and diversity in the gut microbiota of these monkeys in relation to their developmental stages with the group average potentially reflecting the individuals' microbial constituents. No significant difference in the composition within each of the three aged groups of Shennongjia golden snub-nosed monkeys was revealed by all five alpha diversity indices (Figure 1). This may be explained by the rearing approach of these monkeys. At the wildlife reserve of the Hubei Conservation and Research Center for the Golden Monkey in the Shennongjia National Nature Reserve, these monkeys are reared in the wild but fed three times with the same foods each day by breeders at the Center, suggesting that these monkeys contain the same or similar microbial community composition in their gut microbiome. These results are consistent with those reported previously. For example, studies have shown that the gut microbial communities were clustered strongly by diet, i.e., the gut microbial communities were similar in monkeys fed with the same foods, while the phylogenetic relationships among the colobines were not recognized in gut microbiota analyses [20], even though it is generally challenging to distinguish the effects of diet and phylogeny. The lack of significant difference in the alpha diversity indices was also probably due to the small sample size in our study. The sample size, i.e., six individuals included in each of the three aged groups of Shennongjia golden snub-nosed monkeys, is generally smaller than those of similar studies of gut microbiome of snub-nosed monkeys. It is suggested that more individuals of these monkeys should be included in future studies in order to identify the significant differences in these alpha diversity indices. Moreover, the significant difference among these three aged groups was revealed by the beta diversity analysis, showing the association between the microbial community composition and the developmental stages (Figure 6(b)). Similar observations have also been reported in a few species of snub-nosed monkeys [3, 19–21].

### 4.2. Taxonomic Composition of Gut Microbiota in Sichuan Golden Snub-Nosed Monkey

Our results showed that the top 10 most abundant microbial phyla in three aged groups of Shennongjia golden snub-nosed monkeys identified by the high-throughput sequencing included Firmicutes with the highest relative abundance, followed by Bacteroidetes, Verrucomicrobia, Spirochaetes, Tenericutes, Proteobacteria, Planctomycetes, Fibrobacteres, Cyanobacteria, and Euryarchaeota (Table 1). These results are consistent with those reported previously. For example, as the top two most abundant bacterial phyla revealed in our study (Figure 2 and Figure S2), both Firmicutes and Bacteroidetes were also identified as the most abundant phyla in the gut microbiota of human and a few nonhuman primates [26, 36] and other three taxa of snub-nosed monkeys, including black snub-nosed monkeys (also known as the Yunnan snub-nosed monkeys) [19], Sichuan golden snub-nosed monkeys [3], and Guizhou snub-nosed monkeys [21]. In our study, phyla Firmicutes and Bacteroidetes accounted for ~55% of the microbial community compositions with the phylum Firmicutes showing 10~29% lower than those reported previously in the same species and the nonhuman primates [21, 36], while the proportions of other nine phyla showing low relative abundance varied within comparable ranges between our results and those reported previously in the nonhuman primates [36]. It is worth noting that phyla Firmcutes and Bacteroidetes showed opposite patterns in the relative abundance between Shennongjia and Sichuan golden snub-nosed monkeys with the latter containing less Firmcutes and more Bacteroidetes in their gut microbiota [3]. However, the Sichuan golden snub-nosed monkeys with diarrhea showed the similar relative abundance (i.e., more Firmcutes and less Bacteroidetes) as that of the Shennongjia golden snub-nosed monkeys as presented in the current study. Furthermore, in addition to phyla Firmicutes and Bacteroidetes, another two phyla (i.e., Actinobacteria and Spirochaetes) were also identified as the most abundant bacterial phyla in the Chinese-origin rhesus macaques [37]. This distinction of the most abundant microbial phyla in the gut microbiota between Shennongjia golden snub-nosed monkeys and other nonhuman primates is probably caused by the dietary foods fed to Shennongjia golden snub-nosed monkeys. Further studies are necessary to explore the roles played by these two phyla in the health of these monkeys.

The relative abundance of the 18 fecal samples at both the genus and phylum levels was evaluated based on the taxonomic units assigned by the RDP classifier. A total of 35 most abundant microbial genera were revealed in all three aged groups of Shennongjia golden snub-nosed monkeys with varied relative abundances (Figure 3). The varied proportions of these genera in three aged groups of monkeys indicated that the colonization dynamics of the gut microbiota in Shennongjia golden snub-nosed monkeys takes place within a wide range of different developmental stages. Results also showed that significant differences were identified among various genera and phyla of the gut microbiota among the three groups of Shennongjia golden snub-nosed monkeys (Tables 2 and 3). For example, the numbers of OTUs in genera *Mucispirillum* were significantly different between Groups 1 and 2, four genera (i.e., *Escherichia, Anaeroplasma, Clostridium*, and *Flexispira*) were significantly different between Groups 1 and 3, while Groups 2 and 3 were significantly different in the relative abundance in genera *Ruminococcus*, *Anaerostipes*, and *Campylobacter*. Significant differences were also revealed among these three groups at the phylum level. Specifically, phyla Deferribacteres, Planctomycetes, and Lentisphaerae were significantly different between Groups 1 and 2, Groups 1 and 3, and Groups 3 and 2, respectively. These results were consistent with those reported previously indicating that the gut microbiota of Shennongjia golden snub-nosed monkeys sustains its dynamic state during the developmental ages with a few species presenting throughout their lifetime. This pattern has also been reported in Sichuan golden snub-nosed monkeys showing the dynamic change of the microbial community compositions and structures in their gut microbiome [3, 19].

The results of the beta diversity analyses using UPGMA UniFrac clustering (Figure 6) and PCoA (Figure 7) showed that Groups 1 and 2 contained the similar gut microbial community compositions but different from that of Group 3 (containing six individuals above 12 years). The results of species relative abundance (Figure 5) indicated that Group 3 contained the largest and the most diverse groups of OTUs (1,931) in comparison to Group 1 (1,469) and Group 2 (1,381). Furthermore, two of the top 10 most abundant phyla (Planctomyctes and Spirochaetes; Figure 4) and two of the top 35 most abundant genera (*Treponema* and *Anaeroplasma*; Figure 2(b) and Figure S2) showed evidently declined proportions in the three groups of Shennongjia golden snub-nosed monkeys associated with their developmental ages. These results suggest that the bacterial species relative abundance in the gut microbiota increases as the developmental ages of these Shennongjia golden snub-nosed monkeys but not showing evident variations at the young ages. The similar patterns have been revealed in the gut microbiome of Yunnan snub-nosed monkeys through with different genera [19, 40]. For example, *Clostridium* was overrepresented in phylum Firmicutes likely responsible for lignocellulosic biomass degradation [41].

Among the top 10 most abundant microbial genera revealed in the gut microbiome of Shennongjia golden snub-nosed monkeys, four (*Lactobacillus*, *Oscillospira*, *Ruminococcus*, and *Faecalibacterium*) and two (*Prevotella* and *Bacteroides*) genera belong to phyla Firmicutes and Bacteroidetes, respectively. *Lactobacillus* is generally considered as one of the several most important beneficial bacteria in the gastrointestinal tract [42]. Genera *Lactobacillus* and *Prevotella* were revealed in the gut microbiome of Shennongjia golden snub-nosed monkeys with a proportion of 3.26% and 2.58%, respectively. *Lactobacillus* is generally known as functioning to ferment dietary sugars, while *Prevotella* functions as the substitutes to ferment dietary sugars in primates [36]. These results are consistent with the dietary structure of Shennongjia golden snub-nosed monkeys, suggesting the physiological importance of these bacteria in digesting plant materials in their diet (i.e., diet fermentation) and possible disorders in their gastrointestinal tract upon any alteration of these bacteria in gut microbiota. Furthermore, studies have shown that *Lactobacillus* was identified as abundant in Sichuan golden snub-nosed monkeys with diarrhea but less abundant in healthy and old monkeys, while *Prevotella* was dominant in healthy monkeys [3]. Studies have shown that many beneficial effects of gut microbiota on physiological and immunological changes are associated with species of *Lactobacillus* [43–45]. It has been suggested that the relative abundance of *Bacteroidetes* may be used as an indicator of the intestinal health status [3]. Studies have shown that the relative abundance of *Prevotella* was associated with carbohydrate and simple sugar digestion [46], while *Prevotella* was identified as one of the three most abundant genera (with genera *Akkermansia* and *Bacteroides*) in the gut microbiota of Guizhou snub-nosed monkeys in captivity [21]. More studies based on the comparisons of gut microbial communities between the wild and captive monkeys are needed to clarify the biological significance of both *Lactobacillus* and *Prevotella* particularly in both diet fermentation and association with age as well as physiological changes in Shennongjia golden snub-nosed monkeys. As another representative genus of phylum Firmicutes, *Oscillospira* was identified as one of the most abundant genera in our study, particularly in the oldest Group 3 of Shennongjia golden snub-nosed monkeys. This was also reported in both Sichuan and Guizhou snub-nosed monkeys [3, 21]. *Oscillospira* is a rarely cultivated bacterial genus, commonly found in human gut microbiota and considered as the fiber-digesting bacteria [47], while the high relative abundance of *Oscillospira* was also linked to the constipation in human [48]. The high relative abundance of *Oscillospira* in Shennongjia golden snub-nosed monkeys would be explained by their dietary structures. *Ruminococcus*, generally considered as cellulolytic microbes, was revealed to be significantly more abundant in the younger Groups 1 and 2 but not in the oldest Group 3 of Shennongjia golden snub-nosed monkeys. However, this genus was revealed as dominant in healthy and older individuals of Sichuan golden snub-nosed monkeys [3]. Similarly, the genus *Faecalibacterium* was also identified as dominant in healthy and adult individuals of Sichuan golden snub-nosed monkeys [3] but decreased in the gut microbiota of patients with Crohn's disease [49]. Further studies are needed in order to investigate the detailed dynamic relative abundance of these genera in these monkeys.

As one of the representative genera in the phylum Bacteroidetes, *Bacteroides* was revealed as one of the most abundant genera in our study. *Bacteroides* are commonly found in the human intestine where they form a symbiotic host-bacterial relationship with humans. Because the genus *Bacteroides* accounted for the significant portion of the gastrointestinal microbiota showing a vital role in the maintenance of intestinal health in mammals, the relative abundance of *Bacteroidetes* has been suggested as an indicator for the evaluation of the gastrointestinal health status [3]. Specifically, they assist in degrading and fermenting organic matters (i.e., polysaccharides in plant cell walls) and producing valuable nutrients and energy that the body needs [50]. Furthermore, several species in the genus *Bacteroides* play important roles in the degradation of both pectin and lignin containing high nutritional values [51]. *Bacteroides* was also constantly revealed as a dominant genus in Yunnan snub-nosed monkeys [19], Sichuan golden snub-nosed monkeys [3], and Guizhou snub-nosed monkeys [20]. These results evidently demonstrate the dominant status of *Bacteroides* in gut microbiome in maintaining animal health and symbiotic relationships with their hosts.

Our results showed that Proteobacteria was one of the most abundant bacterial phyla with a relative proportion of ~1.2% in the gut microbiota of Shennongjia golden snub-nosed monkeys. Proteobacteria was also identified as a common and dominant phylum in gut microbiome of both human [52] and Yunnan snub-nosed monkeys [3]. The phylum Proteobacteria contains numerous pathogenic bacterial species, e.g., *Escherichia coli*, *Salmonella*, *Vibrio cholerae*, and *Helicobacter pylori*, contributing to various types of diseases in human [53–56]. Therefore, it is speculated that the bacterial species of the phylum Proteobacteria in the gut microbiota of Shennongjia golden snub-nosed monkeys may be related to the bowel diseases. It is worth noting that the individual sample #18 (one of the oldest monkeys above 12 years) showed extremely high relative abundance of Proteobacteria in comparison to other 17 samples. Currently, it is not known whether the relative abundance of the phylum Proteobacteria is associated with the developmental ages based on only one sample. Future studies based on the comparisons of the gut microbiome between healthy and unhealthy monkeys and a more comprehensive sampling are necessary to provide evidence to either support this speculation or to confirm that the phylum Proteobacteria represents the normal composition of the gut microbiota in Shennongjia golden snub-nosed monkeys.

As one of the top 10 most abundant bacterial phyla, Fibrobacteres maintained a proportion of ~0.83% in the gut microbiota of Shennongjia golden snub-nosed monkeys. Fibrobacteres (e.g., *Fibrobacter*) are usually found in the rumens of ruminants functioning to degrade cellulose, thus increasing the nutrient absorption in their hosts [57]. Members of Fibrobacteres are found not only in the mammalian intestinal tract but also in the intestines of termite providing carbon for its host from degrading cellulose [58]. The genus *Fibrobacter* was identified as one of the top 10 most abundant genera in Shennongjia golden snub-nosed monkeys. Similarly, the Yunnan snub-nosed monkeys were also shown to have an overrepresentation of phylum Fibrobacteres based on the metagenomic comparative analysis [19]. These results suggest that the large proportions of phylum Fibrobacteres in Shennongjia golden snub-nosed monkeys are the adaptive characteristics in relation to their dietary structures.

Another most abundant bacterial phylum Spirochaetes was revealed to contain a proportion of ~6% in the gut microbiota of Shennongjia golden snub-nosed monkeys. The Spirochaetes contain numerous pathogenic species including *Leptospira*, *Borrelia burgdorferi*, *B. recurrentis*, *Treponema carateum*, *T. pallidum*, and *T. pertenue* [59]. It is noted that Spirochaetes was not an abundant phylum in human gut microbiota, while the comparative analysis showed that Yunnan snub-nosed monkeys were revealed to show the overrepresentation of Spirochaetes in their gut microbiome [19]. Our results showed that the proportion of genus *Treponema* reached ~3.08–15.05% in gut microbiota of Shennongjia golden snub-nosed monkeys, suggesting the possible existence of novel species with either beneficial or detrimental effects to the gut health of their host. The effects of the change of Spirochaetes proportion in the gut microbiome of Shennongjia golden snub-nosed monkeys on the gut health remain unclear. However, the genus *Treponema* was revealed as abundant in healthy Sichuan golden snub-nosed monkeys [3]. Further studies are needed to confirm the biological functions of *Treponema* in Shennongjia golden snub-nosed monkeys and further resolve these inconsistent observations on these microbial community compositions among these phylogenetically related monkeys.

Phylum Verrucomicrobia generally live in feces of animals as well as water and soil. The proportion of Verrucomicrobia in the gut microbiota of Shennongjia golden snub-nosed monkeys reached ~3.6%. The high proportion of Verrucomicrobia warrants further studies to investigate the biological functions of these bacteria in the gut microbiome of Shennongjia golden snub-nosed monkeys. As one of the representative genera of phylum Verrucomicrobia, *Akkermansia* was identified as one of the most abundant genera in our study. This genus was also identified as one of the dominant genera in Guizhou snub-nosed monkeys in captivity [21]. *Akkermansia*, as the mucin degrader, is generally considered as the biological marker indicating the healthy conditions in the intestines [60] showing increased relative abundance during malnutrition in its hosts [61]. However, the increased relative abundance of *Akkermansia* would be indicating the poor nutrition in the hosts, i.e., the Guizhou snub-nosed monkeys [21]. Further studies are needed to verify the explicit functions of *Akkermansia* in Shennongjia golden snub-nosed monkeys.

### 4.3. Limitations

We note that there are several limitations in our study. These limitations further help us determine the explicit studies on the gut microbiota in the future in Shennonjia golden snub-nosed monkeys. First, this study lacks the samples of unhealthy monkeys to perform a comparative analysis of the gut microbiome between healthy and unhealthy monkeys. During the sample collecting processes, we did not observe any unhealthy monkeys. Furthermore, because the leaf-eating Colobine monkeys are difficult to maintain in captivity and frequently show gastrointestinal diseases, it is expected that more individuals of these monkeys including both the wild and captive animals should be included in the future studies in order to identify the significant differences among the alpha diversity indices. Second, it is generally accepted that the fecal samples may not represent the entire microbial community composition and diversity of the gastrointestinal tract. A more comprehensive sampling through the entire gastrointestinal tract is necessary to obtain more complete data of the microbial community composition and diversity in these monkeys. Third, although the golden snub-nosed monkeys are generally leaf eaters and consume a large amount of plant materials, they are also observed to consume lichens. Due to the structural components of lichens containing a large amount of bacteria, it is important to investigate the contribution of the lichens to the bacterial diversity of gut microbiota in these monkeys. Lastly, the gut microbiome is affected not only by diet [37] but also by the stress, disease, and the microbial environments in captivity [38]. Furthermore, the alteration of the gut microbial composition in Shennonjia golden snub-nosed monkeys could be attributed to the generally healthy aging as well [30]. It is beyond the scope of this study to investigate the functions and interactions of microbial community as well as the metabolic environment of the gastrointestinal tract, which are generally considered as casting stronger effects on host health and disease in comparison to microbial composition.

## 5. Conclusions

A total of 28 microbial phyla were identified in the gut microbial communities of Shennongjia golden snub-nosed monkeys with Firmicutes and Bacteroidetes being the top two most abundant phyla in all three aged groups of monkeys. Groups 1, 2, and 3 were revealed to contain a total of 1206, 1196, and 1618 OTUs in their gut microbiome, respectively. These results suggest that the gut microbiota in these monkeys are in a dynamic status, formed at the early developmental stages of life with the species relative abundance increased as the developmental ages. The thorough understanding of the dynamic interactions between Shennongjia golden snub-nosed monkeys and their gut microbial community compositions is vital for the health and population development of these monkeys. The maintenance of a dynamic balance between hosts and their gut microbiota across different ages would provide critical new perspectives on mammalian ecology and evolution as well. Future studies of gut microbiota in golden snub-nosed monkeys from different geographic locations and other nonhuman primates would help us determine the associated influences of ecology and phylogeny in shaping the gut microbiota of these endangered species. Our study provides a key database of the gut microbial community composition based on Shennongjia golden snub-nosed monkeys of different ages. This important database has set up the scientific foundation and framework for future comparative studies on their digestive adaptivity based on the gut microbiota in these endangered and phylogenetically closely related monkeys in *Rhinopithecus*. A comprehensive characterization of gut microbiota in both captive and wild monkeys provides valuable insights into the effects of captivity on the maintenance or alteration of the gut microbiome, ultimately affecting the health of these animals, particularly the colobine species, which generally fail to thrive in captivity. This information is also important for predicting, monitoring, and improving the health conditions, management, and conservation of Shennongjia golden snub-nosed monkeys based on the deviations in their intestinal microbiome, ultimately protecting this endangered species.

## Figures and Tables

**Table 1:** Proportions of the top 10 most abundant phyla and other phyla (combined) of microbes identified in the three groups of fecal samples of Shennongjia golden snub-nosed monkeys.

Phylum	Group 1	Group 2	Group 3	Average ± SD
Firmicutes	0.560	0.550	0.500	0.537 ± 0.032
Bacteroidetes	0.172	0.203	0.251	0.209 ± 0.040
Verrucomicrobia	0.029	0.036	0.043	0.036 ± 0.007
Spirochaetes	0.081	0.054	0.045	0.060 ± 0.019
Tenericutes	0.029	0.039	0.025	0.031 ± 0.007
Proteobacteria	0.010	0.007	0.019	0.012 ± 0.006
Planctomycetes	0.025	0.009	0.007	0.014 ± 0.010
Fibrobacteres	0.003	0.016	0.006	0.008 ± 0.007
Cyanobacteria	0.012	0.006	0.011	0.010 ± 0.003
Euryarchaeota	0.002	0.001	0.0001	0.001 ± 0.001
Other phyla	0.077	0.079	0.088	0.081 ± 0.005

SD: standard deviation.

**Table 2:** Microbial genera showing a significant difference among three aged groups of Shennongjia golden snub-nosed monkeys identified by both *t*-test in R package and the nonparametric *t*-test in Metastats.

Group pairs	Genus	*P* value/statistical analysis
Group 1 vs. Group 2	*Mucispirillum*	0.0389/*t*-test; 0.0131/Metastats
	*Treponema*	0.0383/Metastats
	*Succinivibrio*	0.0483/Metastats

Group 1 vs. Group 3	*Escherichia*	0.0485/*t*-test; 0.0125/Metastats
	*Anaeroplasma*	0.0197/*t*-test; 0.0067/Metastats
	*Clostridium*	0.0448/*t*-test; 0.0178/Metastats
	*Flexispira*	0.0205/*t*-test; 0.0028/Metastats
	*Treponema*	0.0188/Metastats
	*Ruminococcus*	0.0178/Metastats
	*RFN20*	0.0204/Metastats
	*Butyrivibrio*	0.0325/Metastats

Group 2 vs. Group 3	*Ruminococcus*	0.0412/*t*-test; 0.0135/Metastats
	*Anaerostipes*	0.0071/*t*-test; 0.0030/Metastats
	*Campylobacter*	0.0223/*t*-test
	*Prevotella*	0.0217/Metastats
	*BE24*	0.0427/Metastats
	*Flexispira*	0.0248/Metastats
	*Campylobacter*	0.0087/Metastats

**Table 3:** Microbial phyla showing a significant difference among three aged groups of Shennongjia golden snub-nosed monkeys identified by both *t*-test in R package and the nonparametric *t*-test in Metastats.

Group pairs	Phylum	*P* value/statistical analysis
Group 1 vs. Group 2	Deferribacteres	0.0389/*t*-test; 0.0171/Metastats
	Planctomycetes	0.0363/Metastats

Group 1 vs. Group 3	Planctomycetes	0.0326/*t*-test; 0.0110/Metastats
	Lentisphaerae	0.0441/*t*-test; 0.0153/Metastats
	Spirochaetes	0.0356/Metastats

Group 2 vs. Group 3	Lentisphaerae	0.0357/*t*-test; 0.0122/Metastats

## Data Availability

The data used to support the findings of this study are available from the corresponding author upon request.

## Supplementary Materials

Supplementary Materials Supplementary materials contain the sequencing statistics (Table S1), the rarefaction curves based on plotting the number of operational taxon units (OTUs) with the number of reads and the relative abundance (Figure S1), and relative abundance of the top 10 most abundant microbial phyla and genera (Figure S2) identified in the 18 fecal samples of the Shennongjia golden snub-nosed monkeys.
