# Supplementary material for: Age-Based Variations in the Gut Microbiome of the Shennongjia (Hubei) Golden Snub-Nosed Monkey (Rhinopithecus roxellana hubeiensis)
Source: Biomed Res Int. 2021 Mar 12;2021:6667715. doi: 10.1155/2021/6667715 (PMC7979289; doi:10.1155/2021/6667715)
Supplement: Supplementary Materials — contain the sequencing statistics (Table S1), the rarefaction curves based on plotting the number of operational taxon units (OTUs) with the number of reads and the relative abundance (Figure S1), and relative abundance of the top 10 most abundant microbial phyla and genera (Figure S2) identified in the 18 fecal samples of the Shennongjia golden snub-nosed monkeys. [file 6667715.f1.zip › Figure S1 (1).docx]

Figure S1. Rarefaction curves obtained by plotting the number of operational taxon units (OTUs) with the number of reads (A) and the relative abundance (B) for 18 fecal samples of the Shennongjia golden snub-nosed monkeys.
